# Phosphorylation status of a conserved residue in the adenylate cyclase of *Botrytis cinerea* is involved in regulating photomorphogenesis, circadian rhythm, and pathogenicity

**DOI:** 10.3389/fmicb.2023.1112584

**Published:** 2023-02-15

**Authors:** Yunfei Cai, Xue Chen, Peixuan Li, Weiheng Ren, Qiang Zhang, Yiwen Wang, Yina Jiang, Pinkuan Zhu, Hideyoshi Toyoda, Ling Xu

**Affiliations:** ^1^School of Life Science, East China Normal University, Shanghai, China; ^2^Faculty of Agriculture, Kinki University, Nara, Japan

**Keywords:** *Botrytis cinerea*, adenylate cyclase, cAMP, circadian clock, photomorphogenesis, pathogenicity

## Abstract

Adenylate cyclase (AC) regulates growth, reproduction, and pathogenicity in many fungi by synthesizing cyclic adenosine monophosphate (cAMP) and activating downstream protein kinase A (PKA). *Botrytis cinerea* is a typical necrotrophic plant-pathogenic fungus. It shows a typical photomorphogenic phenotype of conidiation under light and sclerotia formation under dark; both are important reproduction structures for the dispersal and stress resistance of the fungus. The report of *B. cinerea* adenylate cyclase (BAC) mutation showed it affects the production of conidia and sclerotia. However, the regulatory mechanisms of the cAMP signaling pathways in photomorphogenesis have not been clarified. In this study, the S1407 site was proven to be an important conserved residue in the PP2C domain which poses a remarkable impact on the phosphorylation levels and enzyme activity of the BAC and the overall phosphorylation status of total proteins. The point mutation *bac^S1407P^*, complementation *bac^P1407S^*, phosphomimetic mutation *bac^S1407D^*, and phosphodeficient mutation *bac^S1407A^* strains were used for comparison with the light receptor white-collar mutant Δ*bcwcl1* to elucidate the relationship between the cAMP signaling pathway and the light response. The comparison of photomorphogenesis and pathogenicity phenotype, evaluation of circadian clock components, and expression analysis of light response transcription factor genes *Bcltf1*, *Bcltf2*, and *Bcltf3* showed that the cAMP signaling pathway could stabilize the circadian rhythm that is associated with pathogenicity, conidiation, and sclerotium production. Collectively, this reveals that the conserved S1407 residue of BAC is a vital phosphorylation site to regulate the cAMP signaling pathway and affects the photomorphogenesis, circadian rhythm, and pathogenicity of *B. cinerea*.

## Introduction

Adenylate cyclase (AC) is an enzyme that converts ATP into cAMP, and the product cAMP is an important second messenger in eukaryotes. By combining with cAMP, PKA is activated to regulate downstream substrates in response to external environmental signals. Studies on AC, represented by human beings, show that a large number of hormone receptors activated are related to the synthesis of second messenger cAMP (cAMP) by membrane-bound adenylate cyclase. The cAMP signal transduction mediates a wide range of cellular responses and participates in the regulation of cardiac contraction, insulin secretion, and neurotransmitter release (Gloerich and Bos, 2010). In recent years, the most detailed three-dimensional structure diagram of AC has been obtained by analyzing the AC-G protein dimer using freeze electron microscopy, which explains the regulation and self-regulation mechanism of cAMP synthesis (Korkhov and Qi, 2019). In addition, the study on plant growth hormone receptor TIR1/AFBs shows that they have not only E3 ubiquitin ligase activity but also adenylate cyclase activity, and AC activity is crucial for auxin-induced transcriptional regulation. This study proves that the auxin receptor TIR1/AFBs has AC activity which is crucial to its receptor function (Qi et al., 2022). In fungi, the cAMP signaling pathway mediated by AC also attracted extensive research activities due to its association with fungal metabolism, development, reproduction, and pathogenicity (Li et al., 2007).

In our early molecular genetics study with *B. cinerea*, a spontaneously variant mutant strain with defects in pathogenicity, conidiation, and sclerotium reproduction was characterized to be caused by a single nucleotide mutation at the S1407 site of adenylate cyclase (BAC) (Chen et al., 2020), in the PP2C (protein phosphatase, family 2C) domain, which is a negative regulatory domain of protein kinase cascade through dephosphorylation. Besides, BAC contains a Gα binding domain that can bind with upstream G protein; a RAS interacting domain; an LRR domain that can participate in a variety of biological processes; and a catalytic domain of adenosine cyclase type III that catalyzes the transformation of ATP into cAMP (Bassler et al., 2018).

In fungi, studies on AC show that different external signals, like peptidoglycan (PGN), CO_2_, pH, and temperature, can stimulate the activity of AC *via* different domains (Xu et al., 2008). Besides, some studies have shown that some kinds of phosphokinase can phosphorylate AC. In animals, the research of frog erythrocytes shows that the 12-*O*-tetradecanoyl phorbol-13-acetate (TPA), a phorbol ester that activates protein kinase C (PKC), could induce PKC to phosphorylation of the catalytic unit of AC, and that may be involved in the phorbol ester-induced enhancement of AC activity (Yoshimasa et al., 1987). The research of mice shows that the A-kinase-anchoring protein79/150 (AKAPS 79/150) could cluster PKA to phosphorylation AC5 to negative cAMP pathway, and the AKAP79 also could interact with AC6 and AC9 to regulate their activity (Efendiev et al., 2010). In *Saccharomyces cerevisiae* adenylate cyclase Cyr1, Snf1/AMPK could phosphorylate Cyr1 to inhibit its synthesis of cAMP ability and lower PKA activity under low glucose conditions (Nicastro et al., 2015). The above studies show that the phosphorylation level of AC will affect its ability to synthesize cAMP.

Studies on adenylate cyclase of *Schizosaccharomyces pombe* Cyr1 showed that the Gsα domain is the binding site of subunit Gpa2 (Ivey and Hoffman, 2005). In *Candida albicans*, Gpa2 and Gpr1 protein-coupled receptors participate in the response of external amino acid and glucose signals (Maidan et al., 2005a,b), the mutant strains of these two genes showed the defective phenotype of mycelial growth on a solid medium depending on the cAMP signal pathway (Ivey and Hoffman, 2005). In addition, the yeast-double hybridization proved the interaction between Ras1 and the RAS domain, and the mutation of conserved residues in the RAS domain would block the interaction between Ras1 and Cyr1 to affect the cAMP signal pathway (Fang and Wang, 2006). In *S. cerevisiae*, Sgt1 has been shown to affect cAMP signal transduction through direct interaction with the LRR domain of Cyr1 (Dubacq et al., 2002). Moreover, the muramyl dipeptides, a component of peptidoglycan, could activate cAMP synthesis by binding with the LRR domain of Cyr1 (Xu et al., 2008). The above research shows that different external signals regulate the cAMP signal pathway *via* regulatory domains to activate AC domain enzymes. However, the functions and effects of the PP2C domain of AC have rarely been reported.

*Botrytis cinerea* is a necrotrophic fungus that is widely distributed worldwide and can infect more than 1,400 plants (Elad et al., 2016). The diseases caused by *B. cinerea* can occur in both pre and postharvest crops, and huge economic losses are ascribed to this pathogen every year (Dean et al., 2012). *B. cinerea* can form sclerotia and conidia under different light conditions (Schumacher, 2017). Sclerotia can be formed under continuous dark conditions (DD), while the conidia can form under continuous light (LL) and alternation of light and dark (LD) conditions, and microconidia can be formed under continuous dark and low-temperature conditions (Faretra et al., 1988). Therefore, *B. cinerea* can adjust its survival strategy by forming different biological structures to adapt to the external environment in the presence of different light conditions (Hua et al., 2018).

The regulation of growth, pathogenicity, and development is a manifestation of the self-regulation of *B. cinerea* in response to external signals (Hua et al., 2018). Conidia and sclerotia reproduction are photomorphogenesis phenotypes of *B. cinerea* (Canessa et al., 2013; Schumacher et al., 2014; Cohrs et al., 2016; Brandhoff et al., 2017), and changes in light signals during the day induce differences in pathogenicity at different time points in a day (Hevia et al., 2015). The oscillation of the circadian clock components, *B. cinerea frequency* gene 1 (*Bcfrq1*), plays an important role in pathogenesis. In the interaction system between *B. cinerea* and *Arabidopsis thaliana*, the circadian clock of *B. cinerea* is more important for pathogenicity at different time points during the pathogenic process (Hevia et al., 2015). The main pathogenic strategy of *B. cinerea* involves spreading and transmitting diseases *via* the conidia generated under light conditions (Schumacher et al., 2014; Fillinger and Elad, 2016). Therefore, the generation and pathogenesis of conidia have been the focus for controlling *B. cinerea*.

The light-responsive transcription factor (LTF) gene is crucial for responding to different light conditions to regulate conidia and sclerotia reproduction (Schumacher, 2017). The light receptor *Bcwcl1* and three LTF genes, *Bcltf1*, *Bcltf2*, and *Bcltf3*, are the most important components involved in conidia and sclerotia reproduction. The white-collar complex (WCC) can regulate the expression of these genes (Schumacher et al., 2014; Cohrs et al., 2016; Brandhoff et al., 2017). However, as a light receptor and core component of the circadian clock, BcWCL1 does not regulate the expression pattern of light transcription factors under natural light conditions.

Both light and cAMP signals are involved in the formation of conidia and sclerotia in *B. cinerea*. However, the relationship between the *B. cinerea* adenylate cyclase (BAC)–mediated cAMP signaling pathway and the formation of conidia and sclerotia and the circadian clock still needs to be explored. We previously reported that the growth rate, conidia, sclerotia production, and pathogenicity are seriously defective in association with the BAC S1407 site mutation (Chen et al., 2020). In this study, the analysis of the S1407 site of BAC demonstrated that the phosphorylation level of BAC could significantly affect some protein phosphorylation levels, *B. cinerea* pathogenicity at different times of the day, and photomorphogenesis. Furthermore, the circadian clock core components *Bcfrq1*, *Bcwcl1*, and the three LTF genes, *Bcltf1*, *Bcltf2*, and *Bcltf3*, were detected, and the effects of the AC-mediated cAMP pathway on the circadian clock, conidia, and sclerotia reproduction were evaluated.

## Materials and methods

### Bioinformatics

The DNA and protein sequences from *B. cinerea* B05.10 were downloaded from the Ensembl Fungi *Botrytis* database (Amselem et al., 2011; Staats and van Kan, 2012; van Kan et al., 2017).^1^ Other fungal sequences were from the database of the National Centre for Biotechnology (NCBI).^2^ The amino acid sequences of the putative orthologues genes were analyzed using the BlastP algorithm (Altschul et al., 1990) at the NCBI. The functional protein domains of BAC protein were predicted using Interpro and SMART.^3^^,^^4^ Additionally, the phosphorylation site of BAC was predicted using Netphos-3.1.^5^ The analysis of WCC binding sites was conducted according to the previous reports (Froehlich et al., 2003; Wang et al., 2015; Baek et al., 2019).

### Fungal strains and culture conditions

Supplementary Table S1 lists the wild-type and mutant strains of *B. cinerea* used in the present study. The wild type of *B. cinerea* strain B05.10 (Büttner et al., 1994), the BAC single mutant strain *bac^S1407P^*, and the complemented strain *bac^P1407S^* (Chen et al., 2020) were used in the present experiments. The *bac^S1407A^* and *bac^S1407D^* strains were generated in this work by introducing homologous recombination fragments into the B05.10 strain according to the previous method (Schumacher et al., 2012).

All strains of *B. cinerea* were ordinarily cultivated on solid potato dextrose agar (PDA) or complete medium (CM), the PDA medium for strain subculture, and the CM medium were used to avoid unknown nutrients (Pontecorvo et al., 1953). The strains were grown at 23°C using Percival incubators, which were equipped with cool white light fluorescent tubes (light intensity up to 100 μM/m^2^/s; wavelength 400–720 nm) at 12-h intervals of light and dark (12:12-h LD).

The growth rate of the mycelial colony was determined by measuring the increase of the colony diameter per day for each strain growing on the solid medium in a Petri dish. To determine the yield of conidia, the colonies cultured for 14 days were washed twice with 5 ml of water to harvest the conidia suspension, which was subsequently filtered by four layers of gauze to remove the mycelial debris. The conidia suspended in the final filtrate (10 ml) were counted under a hemocytometer.

### Protein phosphorylation

The PP2C and AC domain-coding sequences of BAC and BAC^S1407P^ were isolated from the wild-type and mutant strains and inserted into pET28a+ (Supplementary Figure S4) to produce the BAC-His_6_ PP2C-AC and BAC^S1407P^-His_6_ PP2C-AC vectors, respectively. These vectors were transformed into *Escherichia coli* BL21 cells by a heat-shock method. Target proteins were produced by the addition of 1.5 mM isopropyl β-d-1-thiogalactopyranoside (IPTG) at 30°C. The BAC PP2C-AC domain and BAC^S1407P^ PP2C-AC domain were individually linked to six histamines (His6) and expressed as fusion proteins; eventually, the BAC-His_6_ PP2C-AC domain and BAC^S1407P^-His_6_ PP2C-AC domain fusion proteins were produced and then purified using the Ni-NTA 6FF Sefinose (TM) Resin Kit (Shenggong, Shanghai, China).

For the gel retardation assay, the purified protein was run in SDS-PAGE or mixed with Phos-tag (FUJIFILM Wako Pure Chemical Corporation, Osaka, Japan) to examine the phosphorylation levels of different proteins following the reported method (Gou et al., 2015). In this assay, the phosphate group on the protein binds the Phos-tag with the manganese ion in the gel. Eventually, the relative mass of the protein becomes larger, and therefore, the mobility in the gel becomes slower. The variance of the position of the protein is representative of the phosphorylation levels between different proteins. The fusion proteins were specified by binding with anti-His_6_ and secondary antibody goat anti-mouse IgG HRP (AB-M-M100, GOOD HERE, Hangzhou, China) according to the manufacturer’s instructions. This binding was detected by the Western Blot test reagent dye solution (34,580, Thermo Scientific™, Shanghai, China), where the second antibody was bound to HRP, and the substrate of ECL produced chemiluminescence after being catalyzed by HRP.

For the total protein phosphorylation level test, the mycelia growth on cellophane-covered CM medium for 3 days under light or dark conditions, and the protein extraction buffer (Yang et al., 2013) was used to extract mycelia protein. The anti-phosphoserine antibody (ab9332, Abcam, United Kingdom) was used to analyze the total phosphorylation level.

### Adenylyl cyclase activity assay

The adenylyl cyclase activity assay was performed as described previously with some modifications (Korkhov and Qi, 2019). The reaction mixtures contained 50 mM Tris–HCl, pH 7.5, 150 mM NaCl, 0.1% digitonin, 5 mM MnCl_2_, 10 nM to 100 μM total ATP (Shenggong, Shanghai, China), and 0.01 mg/ml purified BAC. The reactions were started by adding ATP to the protein. The reactions were incubated for 10–30 min at 23°C and were terminated by treatment at 85°C for 10 s. The cAMP content was measured using a highly sensitive ELISA kit (Biosamite, Shanghai, China) according to the manufacturer’s instructions.

### Generation of *Botrytis cinerea* mutants

**Figure 1:**
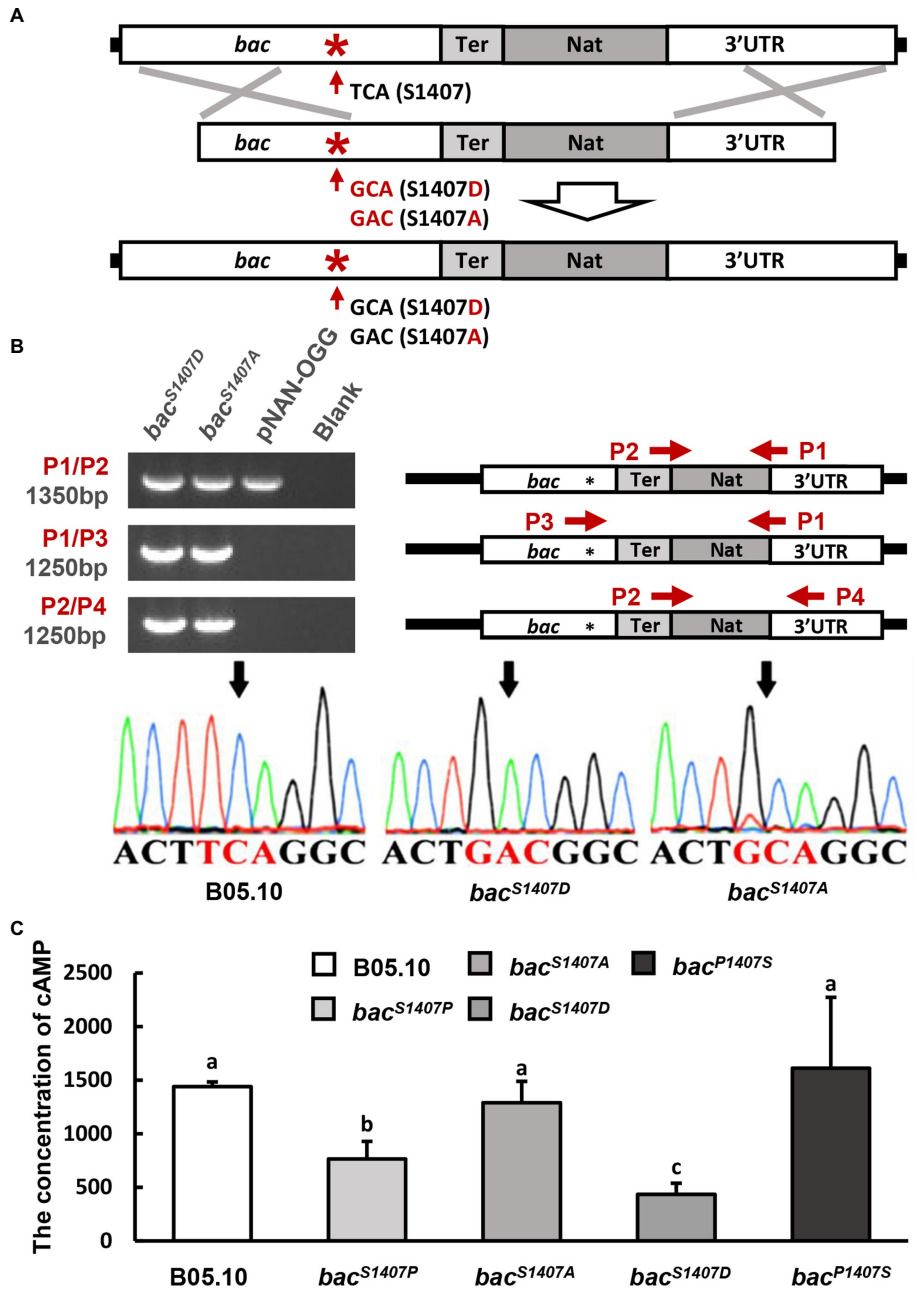
**(A)** Schematic representation of the *in situ* point mutation strategy. **(B)** PCR amplification of target sequences (*bac^S1407D^* and *bac^S1407A^*) by different primer sets (right), and electrophoretic identification of the target sequences (left). **(C)** Intracellular levels of cAMP in the wild type (B05.10), mutant strain (*bac^S1407P^*, *bac^S1407A^*, *bac^S1407D^*, and *bac^P1407S^*) observed after incubation on CM for 3 days under conditions involving light. The different letters on the columns indicate significant differences at *p* < 0.05. The bars present mean values ± SD (*n* = 3).

Mutants of *B. cinerea* were constructed using protoplasts by the method reported previously (Schumacher et al., 2012). The mutant construction strategy is shown in Figure 1A and Supplementary Figure S4. The *bac^S1407A^* and *bac^S1407D^* homologous recombination fragments were constructed by PCR-amplifying their coding regions from chromosomal DNAs using 5′- and 3′-corresponding primers and recombining them with a NAT (nourseothricin resistance cassette) of the pNAN-OGG. These products were transferred to the protoplasts of *B. cinerea* to generate the mutants mentioned above. The pNAN-OGG was used as a template to amplify the NAT fragments. The recombinant sequences were detected by PCR amplification with specific primer sets, as shown in Figure 1A; Supplementary Figure S4. Supplementary Table S2 shows the sequences of these primers.

### Pathogenicity assay

The pathogenicity assay was performed as described previously (Chen et al., 2020). The healthy mature fruits of grape (*Vitis vinifera*), apple (*Malus pumila Mill*), tomato fruit (*Solanum lycopersicum*), 4-week-old tomato leaves, and 2-week-old *A. thaliana* leaves were used in the inoculation assay. Prior to inoculation, the fruits were rinsed in sterile water, submerged in 70% alcohol for the 30 s, and soaked in sterile water for 2 min. The 10 μl droplets of conidial suspensions (1 × 10^6^/ml GB) were dropped onto the inoculation site of the fruit and leaves surface. The inoculated fruits and leaves were placed in a box to keep 90% relative humidity. The lesion area of the inoculation site has been measured *via* Image J software.

### Observation of circadian clock-related phenotype

The banding pattern denotes the alternated, ring-shaped grayish and white regions formed on radially growing mycelial colonies in the presence and absence of light illumination, respectively (Canessa et al., 2013). In order to observe the banding pattern, all strains were incubated for 7 days under LD conditions on the solid CM medium supplemented with 0.02% SDS, which reduces the mycelial growth to approximately 50% of radial growth on the SDS-free medium. This SDS treatment was conducted to make the banding pattern more distinct.

The banding patterns of different strains were further analyzed using a race tube method. The race tube was a transparent cylindrical tube (inner diameter, 9 mm) with air-permeable plugs at both ends. A solid medium was laid inside the tube and placed horizontally, and mycelia clumps of the test strains were separately placed at one end of the tube and incubated at 23°C to examine the change in the banding pattern under different conditions.

To observe the effect of the cAMP signal pathway on the banding pattern, an inhibitor of the cAMP decomposing enzyme PDE, 3-isobutyl-1-methylxanthine (IBMX) (Agarwal et al., 2010) was dissolved in the CM medium with Dimethyl sulfoxide (DMSO) to final concentration 10 μM in solid CM medium and used for the culture of the strains at 23°C under LD condition for a week.

For isolating RNAs from the strains (B05.10, Δ*bcwcl1*, Δ*bcwcl1-com*, and *bac^S1407P^*), they were grown on cellophane membrane-covered PDA plates under the alternation of 12 h-light and 12 h-dark conditions. Samples were harvested in a temperature-controlled (23°C) darkroom equipped with low-intensity red-safety lights. The collected mycelial samples were immediately frozen in liquid nitrogen and subsequently kept at −80°C until further usage.

### Yeast two-hybrid assay

To test protein–protein interactions in yeast cells, the CDS of *BcpkaR*, *Bcwcl1,* and *Bcfrq1* were cloned to pGBKT7 and pGADT7 vectors and then transferred to yeast strain AH109, which has a tryptophan (Trp) and leucine (Leu) deficiency for screening the transformants. Namely, the cells transformed with the constructed pGADT7 and pGBDT7 plasmids were able to grow on the medium lacking His, Ade, Trp, and Leu. In this experiment, the colonies from SD/−Trp/−Leu (-LW) plates were transferred to SD/-Trp//-Leu/-His//-Ade/3-amino-1,2,4-triazole (3-AT) (-LWH/) plates to confirm protein–protein interactions under 28°C for 1 week.

### Yeast one-hybrid assay

To test the binding activity of transcription factors on the promoters of target genes, the CDS of *Bcwcl1* was cloned to pB42AD, and the promoters of *Bcfrq1*, *Bcltf1*, *Bcltf2*, and *Bcltf3* were cloned to pLacZ vector and then transferred to yeast strain EGY48. The yeast was cultured at 30°C until it grew monoclonal at SD/−Trp/-Ura plates. Moreover, the colonies from SD/−Trp/-Ura plates were transferred to SD/−Trp/-Ura plates containing X-gal for culture at 30°C for 2–3 days to confirm the binding activities of transcription factors to promoters, and the combined yeast colonies that can be transcriptionally activated will turn blue.

### RNA extraction and quantitative PCR

For isolating RNAs from the strains, the cellophane membrane-covered PDA plates were used to culture strains. The collected mycelial samples were immediately frozen in liquid nitrogen and subsequently kept at −80°C until further usage. Frozen mycelia were ground to powder in liquid nitrogen, and total RNA was isolated using TRIzol reagent (Invitrogen) as described by Canessa et al. (2013). Total RNA quantity and quality were verified using NanoDrop (Thermo Scientific). All experiments were replicated three times. The expression of different genes in different strains at different times under LD conditions was compared with the expression of 3 h *Bcfrq1* in wild type (B05.10) according to the method described previously (Hevia et al., 2015).

### Statistical analysis

Statistical data were expressed as means ± standard errors (SE) from three repetitions. Bar charts represent mean values with standard deviations, using Tukey’s honestly significant (HSD) test to examine if differences between groups of samples were significant at a *p-*value of <0.01.

## Results

### Changes in BAC phosphorylation levels influence the synthesis of cAMP

**Figure 2:**
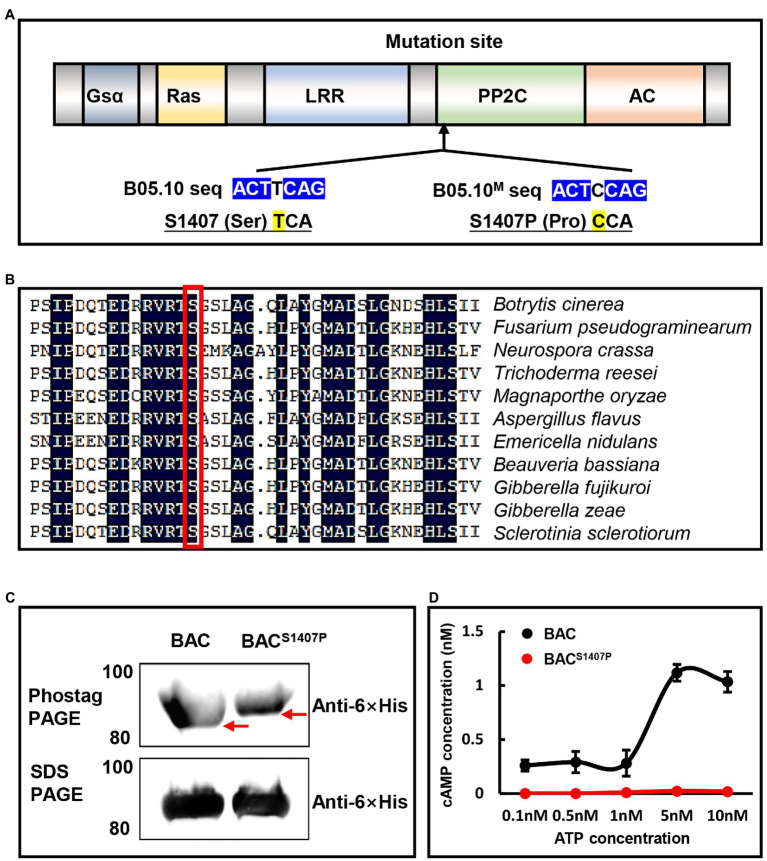
**(A)** *Botrytis cinerea* adenylate cyclase (BAC) domains analyzed using InterPro and SMART. **(B)** Conservation analysis of the S1407 locus of BAC. **(C)** The phosphorylation of BAC-His_6_ and BAC^S1407P^-His_6_ was detected by a mobility shift on a Phos-tag SDS–PAGE gel. Red arrows represent different positions of migration (migration direction from bottom to top). **(D)** Concentration curve of cAMP synthesized by BAC-His_6_ PP2C and AC domains and BAC^S1407P^-His_6_ PP2C and AC domains under different concentrations of ATP.

Our previous study demonstrated that mutation at the S1407 residue in BAC of *B. cinerea* caused obvious defects in growth, development, and pathogenicity (Chen et al., 2020). Here, we used NetPhos-3.1 to predict that the BAC S1407 site was located in the type 2C serine/threonine phosphatases (PP2C) domain of BAC (Figure 2A). The present prediction analysis showed that S1407 was a phosphorylation site of BAC (Supplementary Figure S1). Simultaneously, a comparison of the protein sequences of AC among different fungi by sequence alignment showed that the S1407 was a highly conserved residue (Figure 2B).

To verify the possibility of S1407 as a phosphorylation site, we expressed the BAC-His_6_ PP2C-AC domains (subsequently referred to as BAC) and BAC^S1407P^-His_6_ PP2C-AC domains (subsequently referred to as BAC^S1407P^) using a prokaryotic expression system. Western blot analysis after the protein phosphorylation level assay showed that the position of the BAC band was higher than that of BAC^S1407P^ in SDS-PAGE with Phos-tag; however, the bands were parallel in SDS-PAGE results (Figure 2C). These results indicate that mutations at the S1407 site in the PP2C domain of BAC affected the overall phosphorylation levels of BAC. By adding ATP substrates with different concentration gradients, the change curves of cAMP synthesized by BAC and BAC^S1407P^ were plotted (Figure 2D). The results showed that mutation at the S1407 site of BAC affected the ability of the BAC to convert ATP to cAMP. Taken together, the aforementioned results demonstrate that mutation of the S1407 site in the PP2C domain altered the phosphorylation level of BAC and affected its ability to convert ATP into cAMP.

Replacing the serine residue with alanine can cause this site to dephosphorylate continuously, while mutation to aspartic acid leads to continuous phosphorylation (Kamada et al., 2010). BAC S1407 phosphomimetic strain *bac^S1407D^*, and BAC S1407 phosphodeficient strain *bac^S1407A^*, were obtained by homologous recombination (Figures 1A,B). The intracellular cAMP contents of *bac^S1407P^* and *bac^S1407D^* were lower than those of the wild type, *bac^S1407A,^* and *bac^P1407S^* (Figure 1C). This is consistent with the results observed with the phosphorylation levels of BAC. In addition, the growth rates of the wild-type (B05.10) and mutant strains (*bac^S1407P^*, *bac^S1407D^*, *bac^P1407S^*) were observed in CM medium supplemented with or without exogenous cAMP. The growth rate of *bac^S1407P^* and *bac^S1407D^* was slightly restored by exogenous cAMP (Supplementary Figure S2). After 7 days of culture, the colony morphology of *bac^S1407P^* and *bac^S1407D^* was recovered to some extent (Supplementary Figure S2). These results indicate that the S1407 site of BAC is a phosphorylation site, and the phosphorylation level of this site can affect the BAC activity of cAMP synthesis and mycelial growth.

### BAC PP2C domain S1407 residue mutation affected total protein phosphorylation level

**Figure 3:**
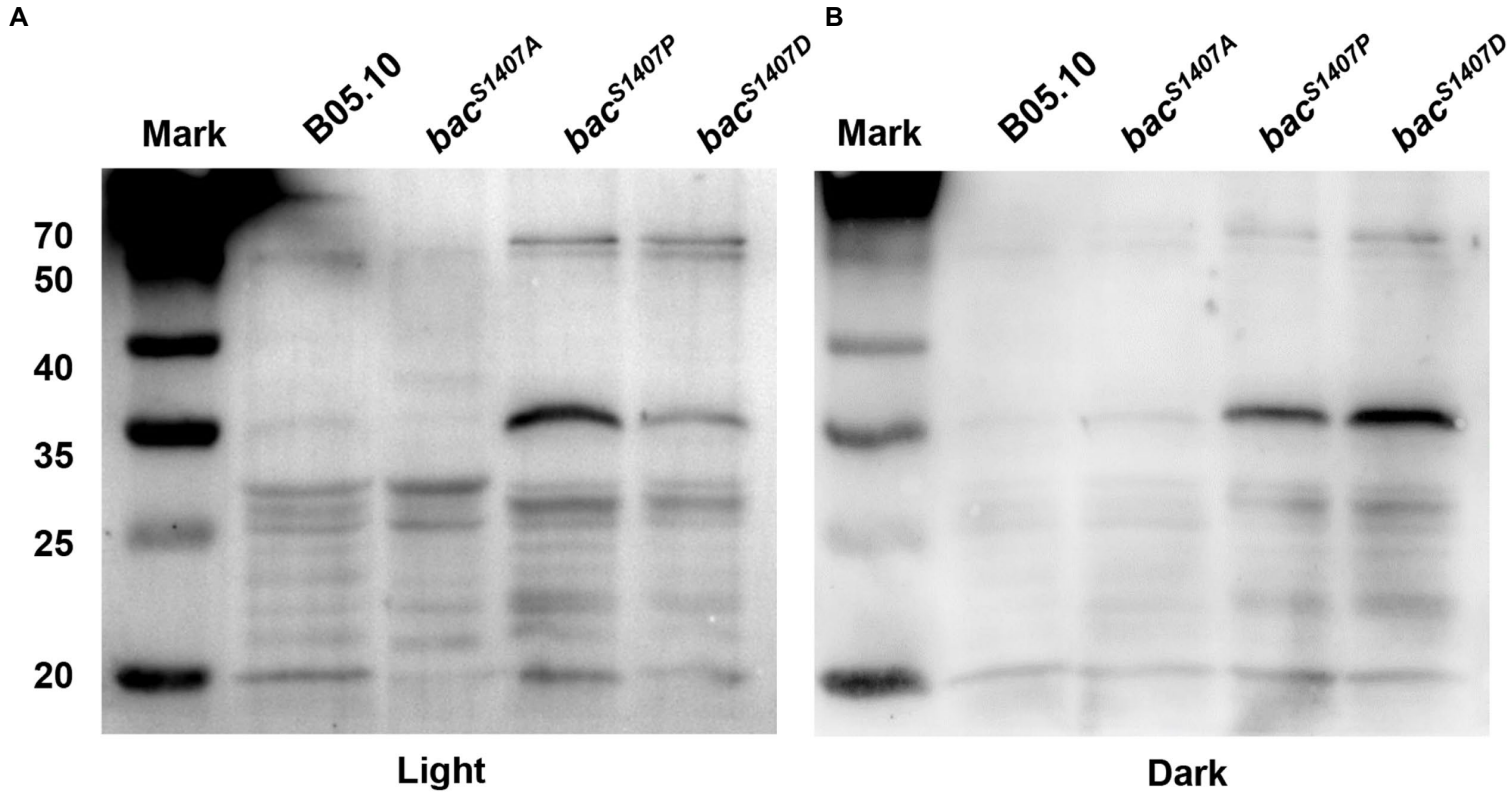
Differences in phosphorylation levels of total proteins of mycelia of wild-type (B05.10) and mutation strains (*bac^S1407P^*, *bac^S1407A^*, and *bac^S1407D^*) growing for 3 days on CM under light **(A)** and dark **(B)** conditions.

PP2C domain is a domain that can remove the phosphates from substrate protein. The mutation of the S1407 site of the PP2C domain of BAC affects the phosphorylation of BAC and the ability of BAC to synthesize cAMP. It may also affect the phosphorylation level of substrate protein of the PP2C domain. We detected the total protein phosphorylation level of strains B05.10, *bac^S1407P^*, *bac^S1407A^,* and *bac^S1407D^* (Figure 3). The results showed that the total protein phosphorylation level of *bac^S1407P^* and *bac^S1407D^* was significantly higher than that of wild type, and the phosphorylation levels of some protein band increased significantly in *bac^S1407P^* and *bac^S1407D^* strains under light and dark conditions (Figure 3), which indicated that the PP2C domain of BAC might have the ability to remove the substrate protein phosphorylation level.

### Phosphorylation levels of BAC are crucial for photomorphogenesis and pathogenicity of *Botrytis cinerea*

**Figure 4:**
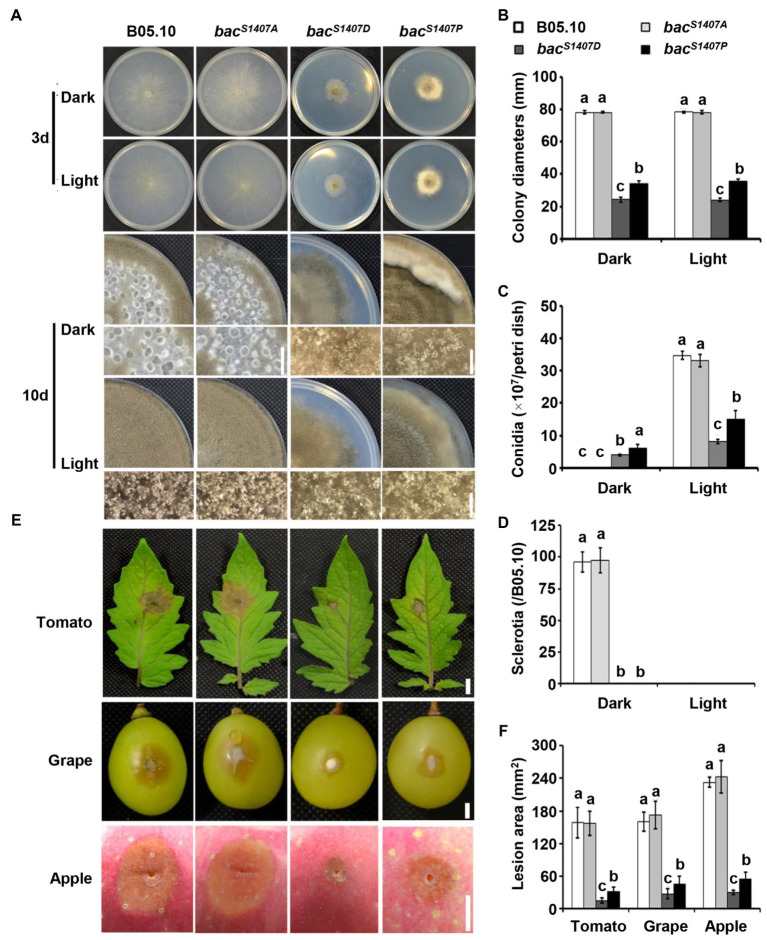
Growth and pathogenic phenotype analysis of different strains. **(A)** Wild-type (B05.10) and mutant strains (*bac^S1407P^*, *bac^S1407A^*, *bac^S1407D^*, and *bac^P1407S^*) were incubated onto solid CM medium for 3 or 10 days under continuous white light (LL) and dark (DD) conditions. **(B–D)** Statistical analyses of the colony diameters, conidia, and sclerotia of **(A)**. **(E)** Wild-type (B05.10) and mutant strains (*bac^S1407P^*, *bac^S1407A^*, *bac^S1407D^*, and *bac^P1407S^*) were incubated onto grape (72 h), tomato leaf (48 h), and apple surfaces (96 h). **(F)** Statistical analysis of the lesion area of **(E)**. Different letters indicate significant differences at *p* < 0.01. The bars present mean values ± SD (*n* = 3).

Due to mutation at the S1407 residue, the phosphorylation level and function of BAC are greatly affected. The growth rate of *bac^S1407D^* was significantly lower than that of the wild type and even lower than that of *bac^S1407P^*. In addition, *bac^S1407D^* mutant continuously produced conidia under LD conditions (Figures 4A–D). The pathogenicity and response to light of *bac^S1407D^* were weaker than those of *bac^S1407P^*, while the pathogenicity, conidiation, and sclerotia production and light responses of the *bac^S1407A^* mutant were similar to those of the wild type (Figures 4E,F). The above results prove that the phosphorylation level of BAC can regulate the production of conidia and sclerotia. The light signaling pathway is also involved in regulating the development of conidia and sclerotia. We compared the conidia-producing phenotype of the Δ*bcwcl1* mutant with the BAC S1407 site mutant strains and found that the yield of conidia increased in Δ*bcwcl1* but decreased in *bac^S1407P^* and *bac^S1407D^* (Supplementary Figure S2C). The cAMP signaling pathway of *B. cinerea* may be involved in the regulation of photomorphogenesis, which is crucial for the formation of conidia and sclerotia of *B. cinerea*.

**Figure 5:**
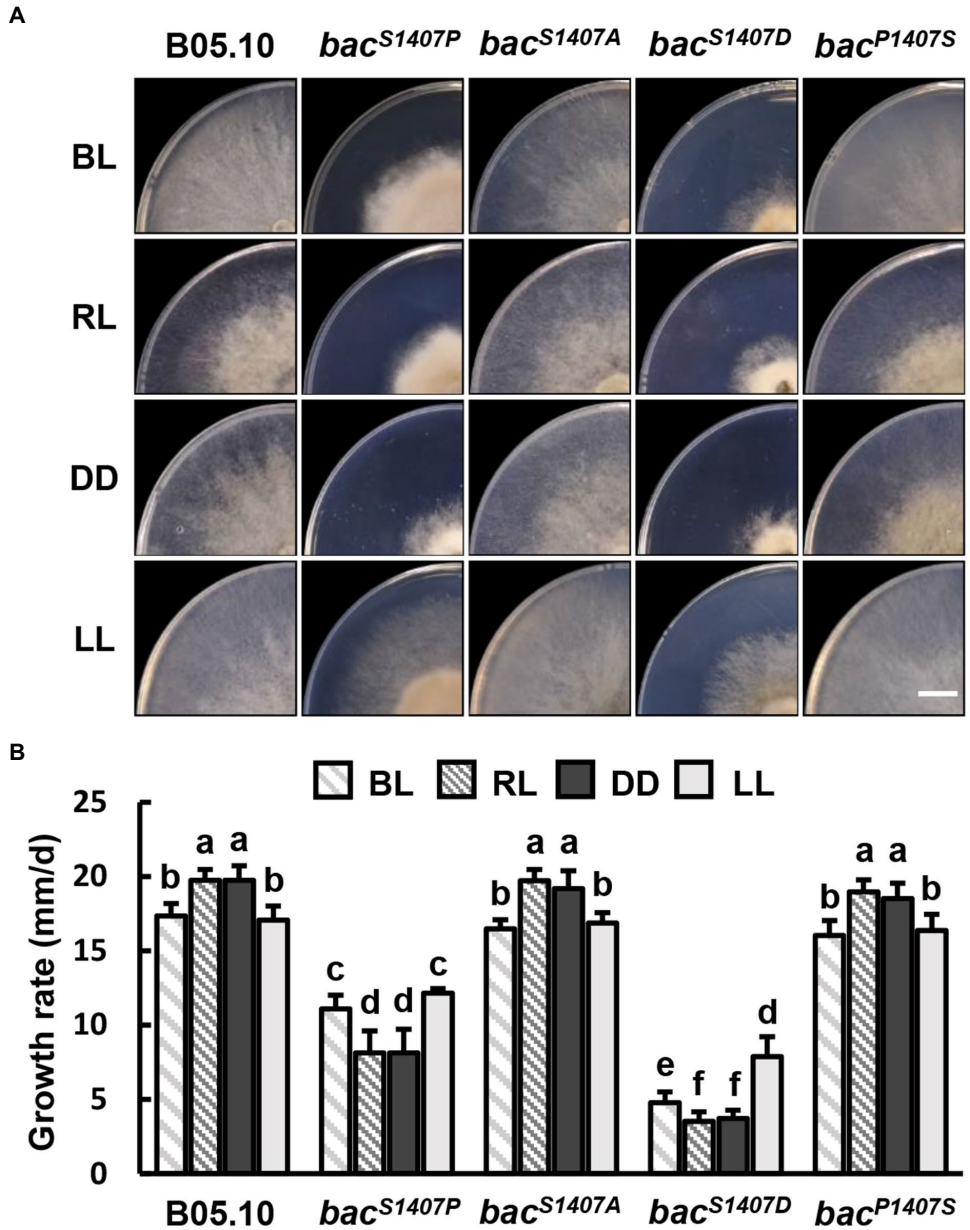
**(A)** Colony appearance of different strains under blue light (BL), red light (RL), continuous dark (DD), and continuous light (LL) conditions. Wild-type (B05.10) and mutant strains (*bac^S1407P^*, *bac^S1407A^*, *bac^S1407D^*, and *bac^P1407S^*) were incubated onto the solid CM medium for 3 days under BL, RL, DD, and LL conditions. The white bar represents 10 mm. **(B)** The colony growth rate of test strains under the same conditions as those depicted in **(A)**. Different letters indicate significant differences at *p* < 0.05. The bars present mean values ± SDs (*n* = 3).

We further explored the effects of varied site mutations of BAC S1407 on growth rate under different light conditions. The colony appearances of different strains grown on CM medium under Blue light (BL), Red light (RL), DD, and LL conditions are shown (Figure 5A). The growth rates of *bac^S1407P^* and *bac^S1407D^* were significantly lower than those of the wild type under BL, RL, LL, and DD (Figure 5B). Interestingly, the growth rates of *bac^S1407P^* and *bac^S1407D^* under BL and LL conditions were higher than those of RL and DD (Figure 5B), suggesting that cAMP participates in mediating the response to light in *B. cinerea*.

### Increases in the phosphorylation levels of BAC intensify the differences in pathogenicity between *Botrytis cinerea* at dawn and dusk

**Figure 6:**
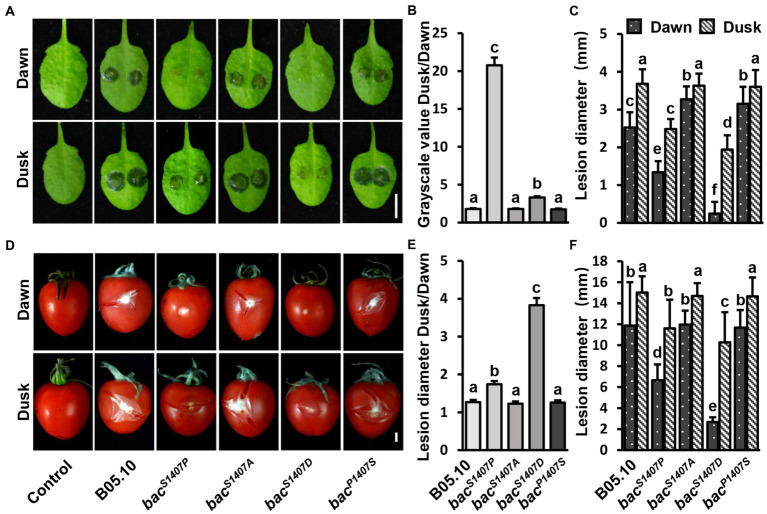
Comparison of lesion areas at dawn and dusk during the interaction of *B. cinerea* and *A. thaliana* leaves **(A)** and *B. cinerea* and tomato fruits **(D)**. The conidia were cultured under LD conditions. PDB was used to suspend the conidia to 1 × 10^6^ conidia/μl, the suspension was then placed in the dark for 24 h after inoculation. The white bar represents 5 mm. **(B,E)** Statistical analysis of contrast of gray values of the lesions recorded at dusk and dawn in **(A,D)**. **(C,F)** Statistical analysis of lesion diameter in **(A,D)**. Different letters indicate significant differences at *p* < 0.05. The bars present mean values ± SDs (*n* = 3).

The conidia of *B. cinerea* are important propagules for infection and transmission, and their formation is regulated by light (Williamson et al., 2007). A study on the interaction between *B. cinerea* and *A. thaliana* showed that the pathogenicity of *B. cinerea* was significantly higher at dusk than at dawn, and this phenomenon is regulated by the circadian clock of *B. cinerea* (Hevia et al., 2015). In the *bac^S1407P^* strain, the yield of conidia was significantly affected, and the ability to produce sclerotia was lost. The conidia of wild type (B05.10) and BAC with S1407 site mutations (*bac^S1407P^*, *bac^S1407A^*, *bac^P1407S^*) were inoculated on *A. thaliana* leaves (Figures 6A–C) and tomato fruits (Figures 6D–F). The pathogenicity of wild-type, *bac^S1407A^*, and *bac^P1407S^* conidia was significantly different at dawn and dusk (Figures 6A,D). However, the differences in pathogenicity between *bac^S1407P^* and *bac^S1407D^* at dawn and dusk were significantly greater than those of the wild type (Figures 6B,C,E,F). These results indicate that the BAC-mediated cAMP signaling pathway is involved in the regulation of differences in the pathogenicity of *B. cinerea* between dawn and dusk.

### Mutations in the phosphorylation site of PP2C domain in BAC significantly influence the circadian growth rhythm of *Botrytis cinerea*

**Figure 7:**
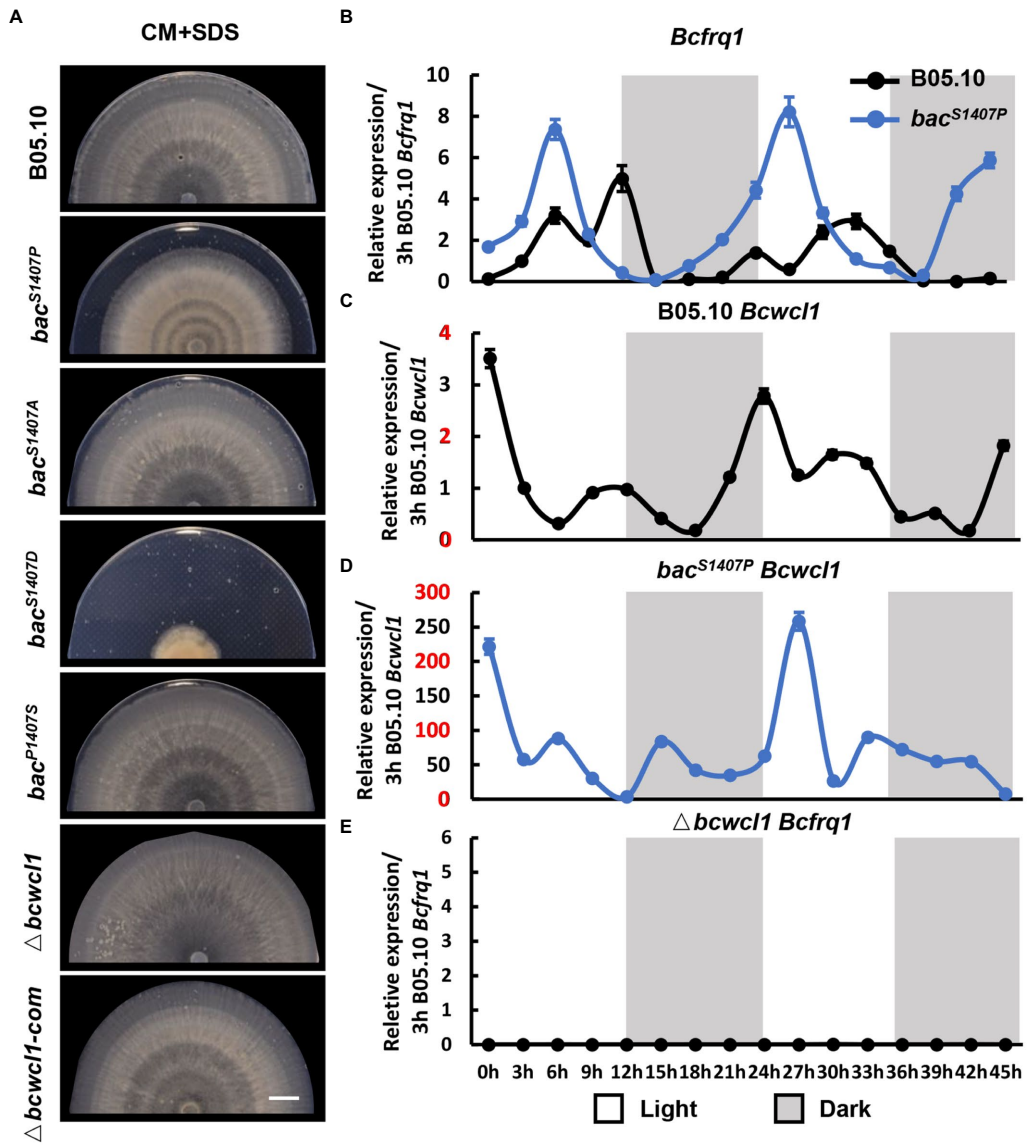
**(A)** The banding pattern of the mycelial colony produced by the wild type (B05.10) and mutant (*bac^S1407P^*, *bac^S1407A^*, *bac^S1407D^*, *bac^P1407S^*, Δ*bcwcl1*, and Δ*bcwcl1-com*) strains on SDS-containing CM medium for 7 days under LD conditions. SDS-containing CM medium was used to reduce mycelial growth to approximately 50% of ordinary radial growth to obtain a clearer pattern. The white bar represents 10 mm. The expression of circadian clock components *Bcfrq1* in wild-type B05.10, *bac^S1407P^* **(B)** and Δ*bcwcl1* **(E)**, and *Bcwcl1* in the wild type **(C,D)**. The expression of B05.10 *Bcfrq1* at 3 h **(B,E)** and B05.10 *Bcwcl1* at 3 h **(C,D)** was used as a ruler to compare the expression changes of genes at different time points under LD conditions for 2 days. Error bars are standard deviations (*n* = 3).

The light signaling pathway is one of the most common environmental factors that regulate the circadian rhythm of fungi (Hevia et al., 2016). Under alternating light and dark conditions, fungi form a weak ring which is due to different growth states and is recognized as the growth rhythm. This growth rhythm is more apparent if SDS is added to the medium because the mycelium growth rate is reduced (Canessa et al., 2013). We compared the differences in circadian rhythms between the wild type (B05.10) and mutant (*bac^S1407P^*, *bac^S1407D^*, *bac^S1407A^*, and Δ*bcwcl1*) under LD conditions (Figure 7A). The photoreceptor BcWCL1 is sensitive to light and is an important component of the circadian clock. Therefore, Δ*bcwcl1* showed no apparent growth rhythm on the CM supplemented with SDS. The *bac^S1407A^* showed no significant changes compared with the wild type, and the growth of *bac^S1407D^* was seriously affected. Hence, it was difficult to observe the rhythm phenotype. However, the growth rhythm of *bac^S1407P^* was more significant than that of wild-type B05.10 (Figure 7A). This is consistent with the results of circadian rhythm observed in different PDA and CM media without SDS (Supplementary Figure S3). These results indicate that the BAC-mediated cAMP signaling pathway is involved in the operation of the circadian clock.

The 45 h-expression curve of the circadian clock core components *Bcwcl1* and *Bcfrq1* in wild type and *bac^S1407P^* under conditions with light–dark alternation was verified at this point (Figures 7B–E). Transcript levels of *Bcfrq1* and *Bcwcl1* showed rhythmic changes in the wild type (Figures 6B,C). Compared with that in *bac^S1407P^*, the expression of *Bcfrq1* and *Bcwcl1* was significantly higher, and the expression of *Bcwcl1* increased significantly in *bac^S1407P^* (Figure 7D). The rhythm of *Bcfrq1* in *bac^S1407P^* was faster than the wild type, and the expression peak was reduced (Figure 7B). The expression of *Bcfrq1* in Δ*bcwcl1* was significantly decreased without rhythm, which was consistent with its rhythmic phenotype (Figure 7A). This result is consistent with the phenotype of the mutant strains observed at the S1407 site of BAC, with elevated BAC phosphorylation levels contributing to the more significant difference in pathogenicity between dawn and dusk (Figure 6). These results further demonstrate that BAC is involved in the regulation of rhythmic growth and pathogenicity.

### The BAC-mediated cAMP pathway component BcPKAR could interact with circadian clock components

**Figure 8:**
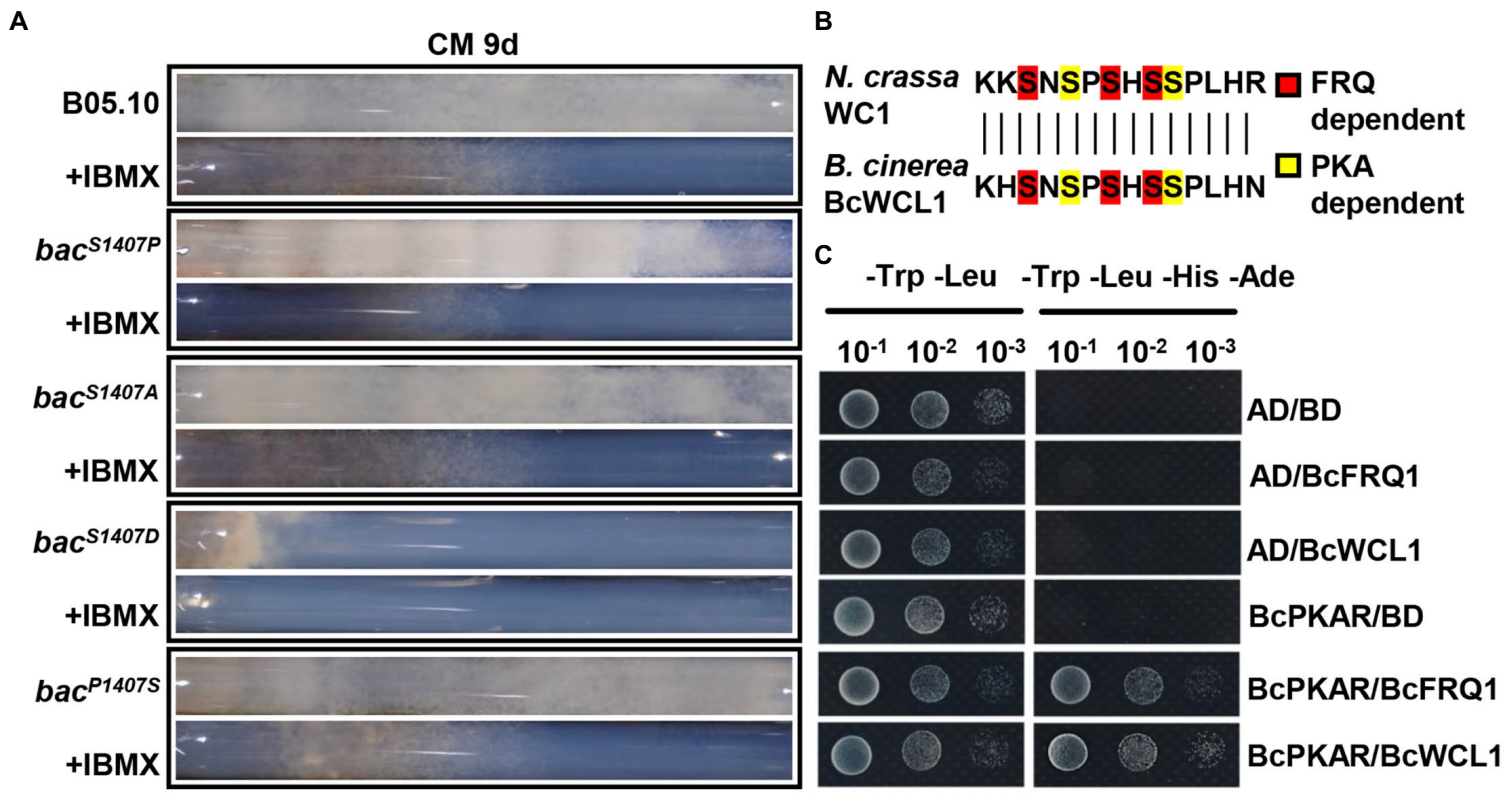
**(A)** Race tube assay for the same strains grown on CM media with or without IBMX at 9 days under LD conditions along with the circadian rhythms after incubation. **(B)** Comparison of amino acid sequences in phosphorylation sites between *N. crassa* WC1 and *B. cinerea* BcWCL. **(C)** Yeast two-hybrid analysis of BcPKAR, BcFRQ1, and BcWCL1. In this assay, yeast cells were used at various densities (10^−1^ to 10^−3^ conidia/μl dilution). AD and BD represent empty plasmids.

Phosphodiesterases (PDEs), which can decompose cAMP, are inhibited by isobutylmethylxanthine (IBMX) for the disruption of the cAMP signaling pathway (Agarwal et al., 2010). The inhibitor IBMX was added to the CM medium in the race tube to verify the role of the BAC-mediated cAMP-PKA signaling pathway in the circadian clock. In the race tube containing CM medium supplemented with IBMX, the banding pattern of *bac^S1407P^* disappeared under LD conditions (Figure 8A). Owing to the lack of operation of the circadian clock, the surface of the mycelial colony of all strains became flattered in comparison to that observed with the IBMX-free CM medium under LD conditions (Supplementary Figure S3B). These results verify the regulatory effect of cAMP on the circadian clock.

Studies in *N. crassa* have shown that PKA can phosphorylate FRQ1 and WC1 (Huang et al., 2007), and nucleotide sequence comparison has shown that the phosphorylation sites of WCL1 are largely conserved in comparison to those in WC1 (Huang et al., 2007; Figure 8B). The cAMP-PKA signaling pathway regulatory subunit BcPKAR can be activated by cAMP to transfer the signal (Liñeiro et al., 2016). The present yeast two-hybrid (Y2H) assay system analysis indicated that BcPKAR interacted with BcWCL1 and BcFRQ1 (Figure 8C). These results suggest that *B. cinerea* may have a similar regulatory mechanism as *N. crassa*.

### BAC participates in influencing the expression mode of the light transcription factor of *Botrytis cinerea*

**Figure 9:**
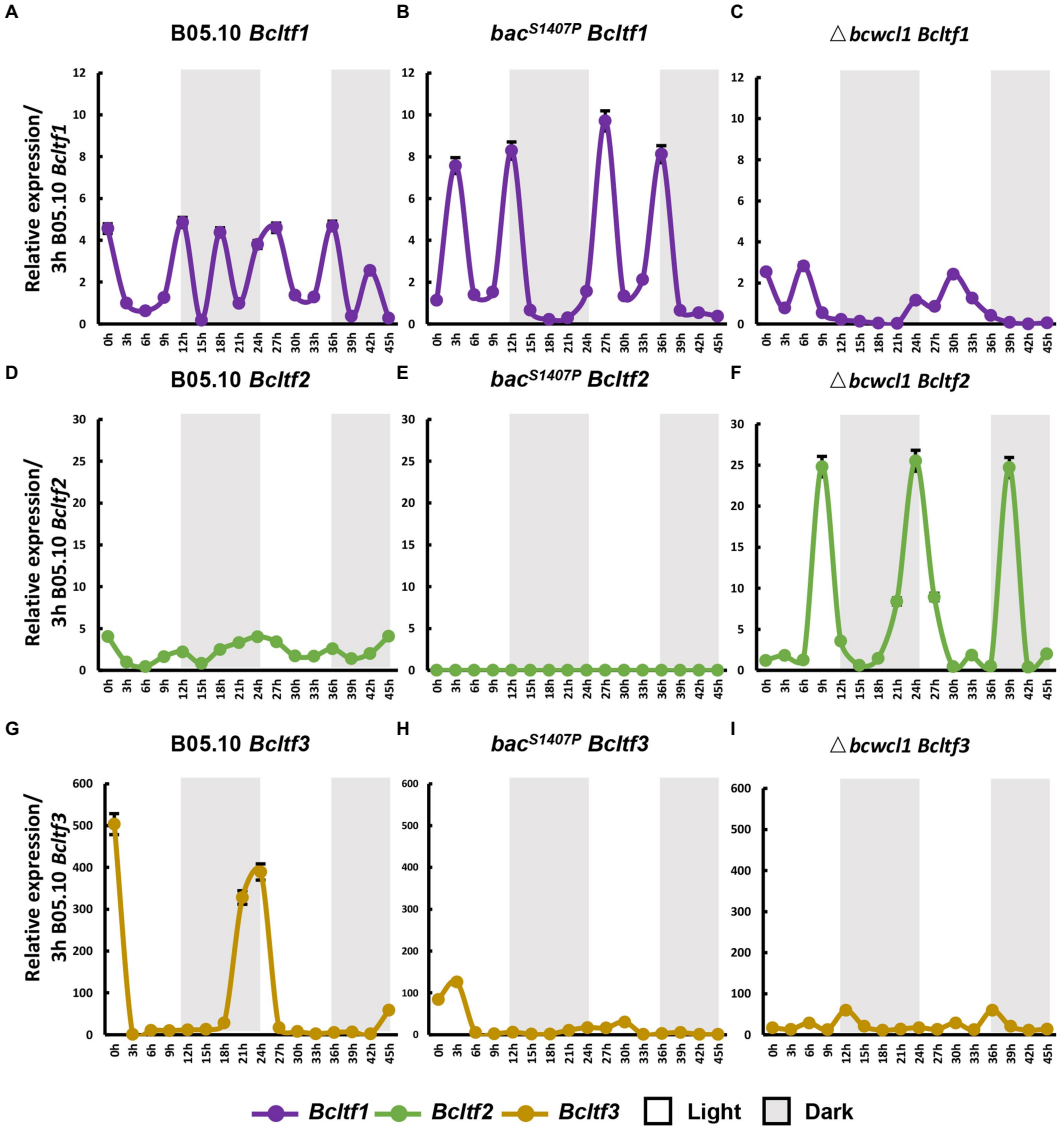
The conidia and sclerotia reproduction-related genes, *Bcltf1*, *Bcltf2*, and *Bcltf3* in the wild type (B05.10) **(A,D,G)**, *bac^S1407P^* **(B,E,H)**, and Δ*bcwcl1* **(C,F,I)** at different time points under LD conditions. The expression of B05.10 *Bcltf1* **(A–C)**, B05.10 *Bcltf2* **(D–F)**, and B05.10 *Bcltf3* **(G–I)** recorded after 3 h was used as a ruler to compare the changes in the expression of genes at different time points under LD conditions for 2 days. Error bars represent standard deviations (*n* = 3).

The phenotypes of BAC S1407 mutations indicate that BAC is crucial to the photomorphogenesis of *B. cinerea* and that *Bcltf1*, *Bcltf2*, and *Bcltf3* are the key transcription regulators downstream of WCC that regulate conidia production under light and sclerotia under dark conditions in *B. cinerea* (Schumacher et al., 2014; Cohrs et al., 2016; Brandhoff et al., 2017). The changes in the expression of *Bcltf1*, *Bcltf2*, and *Bcltf3* in wild type and *bac^S1407P^* observed under LD conditions showed that these three genes were significantly different from those in the wild type (Figure 9). In the wild type, compared with the expression curves of core components of the circadian clock, namely, *Bcfrq1* and *Bcwcl1* (Figures 9B,C), the expression curves of *Bcltf1* and *Bcltf2* showed that they were more remarkably controlled by the circadian clock (Figures 9A,D). However, *Bcltf3* was less sensitive to the clock in the wild type, which was more relevant to light regulation (Figure 9H). The expression oscillation amplitude of *Bcltf1* increased (Figure 9B), and the expression oscillation of *Bcltf3* was significantly reduced (Figure 9I) in *bac^S1407P^*, indicating that the BAC-mediated cAMP signaling pathway has a regulatory effect on it. Unlike the predicted results, the oscillation of *Bcltf2* expression was remarkably reduced, and the rhythm disappeared in *bac^S1407P^* compared to that in B05.10 (Figure 9E). This also explains why the production of conidia sharply declined due to the remarkable reduction in *Bcltf2* expression, which was influenced by the cAMP signaling pathway in *bac^S1407P^*.

### The circadian clock core BcWCL1 can regulate *Bcfrq1* and light transcription factor gene promoters

In *bac^S1407P^*, the expression curve of *Bcwcl1* is significantly higher than that of wild type (Figure 7D), while *Bcfrq1* in Δ*bcwcl1* is almost not expressed (Figure 7E), and the expression of LTF in *bac^S1407P^* and Δ*bcwcl1* is significantly changed compared with that of wild type (Figures 9C,F,J), which indicates that BcWCL1, the core component of the circadian clock, can regulate LTF, but its regulatory mechanism has not been reported.

**Figure 10:**
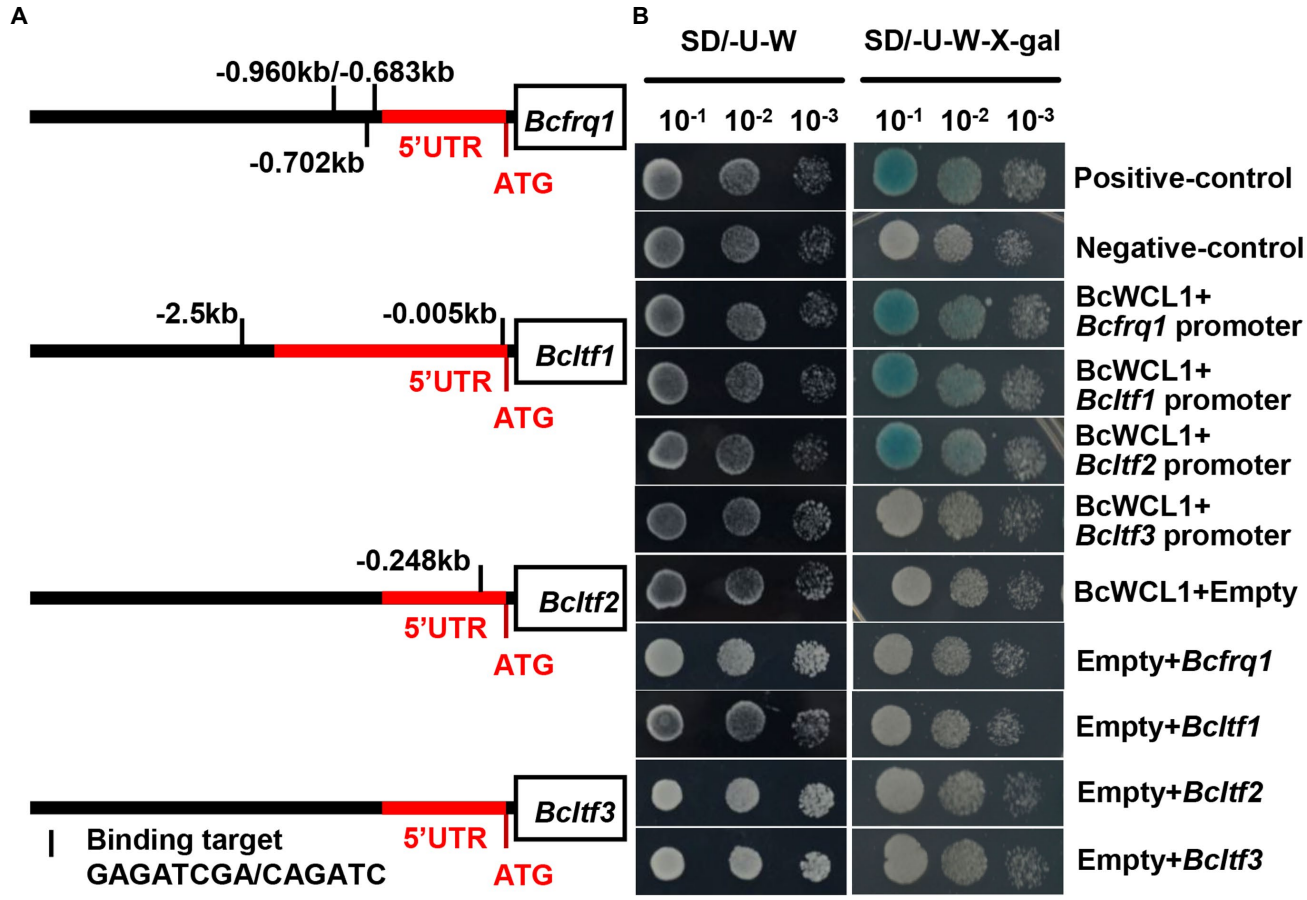
**(A)** WCC binding site analysis of the *Bcfrq1*, *Bcltf1*, *Bcltf2*, and *Bcltf3* promoter regions. The red and black regions represent the gene 5’UTR and the genome region before the 5’UTR, respectively. **(B)** Promoter binding evaluation of BcWCL1 and *Bcfrq1*, *Bcltf1*, *Bcltf2*, and *Bcltf3* by Yeast one-hybrid analysis.

The zinc finger domain of WCC is highly conserved, and the amino acid sequence for DNA binding has been identified in this domain (Wang et al., 2015). Based on the WCC consensus-binding site reported by Baek et al. (2019), the reported WCC downstream gene (*Bcfrq1*, *Bcltf1*, *Bcltf2*, and *Bcltf3*) promoter regions were analyzed. *Bcfrq1* had three putative binding sites on the promoter, whereas *Bcltf1* and *Bcltf2* possessed one putative site (Figure 10A). In contrast, *Bcltf3* did not carry a WCC consensus-binding site (Figure 10A). These results indicate that *Bcltf3* is indirectly regulated by WCC. The yeast one-hybrid assay was used to verify the transcriptional regulation of *Bcfrq1*, *Bcltf1*, *Bcltf2*, and *Bcltf3* in BcWCL1 (Figure 10B). Consistent with the promoter binding site prediction of *Bcfrq1*, *Bcltf1*, *Bcltf2*, and *Bcltf3* in BcWCL1, the yeast one-hybrid assay proved that BcWCL1 could regulate the promoters of *Bcfrq1*, *Bcltf1*, and *Bcltf2*, but not that of *Bcltf3* (Figure 10B). In conclusion, the BAC mediated cAMP signaling pathway can regulate the expression of *Bcwcl1* and LTF to change the survival strategy of *B. cinerea* in the natural environment.

## Discussion

Adenylate cyclase (ACs) in fungi regulate various physiological events during their life cycles. In fact, in *Magnaporthe grisea* (Gushchin et al., 2017), *N. crassa* (Héctor et al., 1974), *Aspergillus flavus* (Yang et al., 2016), *Colletotrichum higginsianum* (Zhu et al., 2017), *Beauveria bassiana* (Wang et al., 2014), and some *Fusarium* species (Li et al., 2018), AC mutations affect mycelial growth, conidia development, and pathogenicity. In addition, the AC mutant *sac1* of *Sclerotinia sclerotiorum* showed a defect in sclerotia development, and the AC mutant *acyA* of *A. flavus* affected sclerotia formation (Klimpel et al., 2002; Lafon et al., 2006). AC mutations that mediate a defect in the production of conidia and sclerotia are conserved in many fungi and affect the regulation of the cAMP signaling pathway. Klimpel et al. (2002) first reported that BAC mutations affect vegetative growth, sporulation, sclerotium production, and pathogenicity. Schumacher et al. (2008) reported that the cAMP-PKA signal pathway is involved in mediating other component functions, and deletion of the PKA regulatory subunit BcPKAR leads to the loss of PKA activity. The Δ*bcpkaR* mutations have a similar photo-morphological defect phenotype as *bac*, which only produces conidia but does not produce sclerotia. Our study showed that the cAMP signaling pathway mediated by AC could stabilize the operation of the circadian clock. Eventually, it ensures that *B. cinerea* can grow hyphae and produce conidia or sclerotia under appropriate light conditions.

Other domains of fungal adenylate cyclase similar to BAC have been reported, but the functions of the PP2C domain in AC have not been sufficiently verified in fungi. The protein family with PP2C as the main domain in fungi is called the PTC family. Studies on the members of this family in *S. cerevisiae* and *C. albicans* have shown that the PTC family is widely involved in the phosphorylation level regulation of different kinases in the fungal MAPK signal pathway (Ariño et al., 2011). Different from *S. cerevisiae*, BcPtc3, but not BcPtc1, negatively regulates phosphorylation of BcSak1 (the homolog of *S. cerevisiae* Hog1) in *B. cinerea*, and both BcPTC1 and BcPTC3 could rescue the yeast PTC1 deletion mutations growth defects phenotype under various stress conditions (Yang et al., 2013). Our study showed that the S1407 site is located in the PP2C domain and is a very important phosphorylation site; the mutation from serine to proline and aspartate at this site can directly affect the phosphorylation level of BAC and the function of cAMP synthesis, whereas the mutation from serine to alanine at this site does not affect the function of BAC, it indicates that the PP2C domain plays a very important role in BAC functions.

Studies on the PP2C family in fungi show that they are widely involved in the regulation of the phosphorylation level of the MAPK signaling pathway in fungi (Ariño et al., 2011). In addition, *Bcptc1* and *Bcptc3* of the PP2C family were proven to be involved in regulating the HOG-MAPK signaling pathway in *B. cinerea* (Yang et al., 2013). In our study, compared with the wild type, the mutation of the S1407 site of the PP2C domain of BAC will increase the phosphorylation level of the total protein and will not be affected by light, and the phosphorylation level of some protein bands increase significantly. Otherwise, the exogenous addition of cAMP did not completely restore the *bac^S1407P^* phenotype (Supplementary Figure S3). The PP2C domain of AC was considered to be involved in substrate dephosphorylation, in addition to the synthesis of cAMP. The functions of the PP2C domain in AC should be further explored in future studies.

It was previously revealed that the results of the interaction between *B. cinerea* and *A. thaliana* varied with the time of day and that the circadian clock of fungi was necessary for maximum pathogenicity at dusk (Hevia et al., 2015). In this study, the *bac^S1407P^* and *bac^S1407D^* mutations in BAC increased the differences in the pathogenicity of *B. cinerea* between dawn and dusk, indicating that the cAMP signaling pathway can also affect the circadian rhythm-regulated pathogenesis oscillation in the fungal pathogen. In nature, the alternation of light and dark is an environmental change experienced by most organisms. Research on the changes in the pathogenicity of plant-pathogenic fungi will help us better prevent and control them.

Compared with the wild type, mutation at the S1407 site of BAC significantly enhanced the circadian rhythm of *bac^S1407P^*, indicating that the cAMP signaling pathway can stabilize the circadian clock component expression. Moreover, compared with the B05.10, two *Bcfrq1* expression peaks in the light phase under LD conditions and the early expression peak of *Bcfrq1* in *bac^S1407P^* under LD conditions were affected, which may imply that BAC-mediated cAMP production participates in the early light response. In addition to these downstream genes, the genes upstream of AC and the external environmental signals that can regulate AC are of great significance for the *B. cinerea* study.

Among the light-responsive transcription factors, *Bcltf1*, *Bcltf2*, and *Bcltf3* are the three most important LTFs for regulating the development of conidia and sclerotia. The white-collar complex (WCC) can regulate the expression of these genes (Schumacher et al., 2014; Cohrs et al., 2016; Brandhoff et al., 2017). In this study, the expression curve of *Bcltf2* in *bac^S1407P^* and Δ*bcwcl1* under LD conditions might explain why conidia production in *bac^S1407P^* was significantly reduced, while that in Δ*bcwcl1* was significantly increased. Compared with the expression curves of *Bcltf1* and *Bcltf2* in wild type and Δ*bcwcl1* under LD conditions, *Bcltf3* was more tightly regulated by light *via* the WCC than that observed with the circadian clock. The analysis of the promoters of BcWCL1 and LTF in the yeast single-hybrid assay verified this finding. This indicates that the mutation at the S1407 site of BAC mediates an increase in BAC phosphorylation levels and the loss of cAMP synthesis ability, ultimately regulating the photomorphogenesis of *B. cinerea* by affecting the expression mode of light transcription factors.

**Figure 11:**
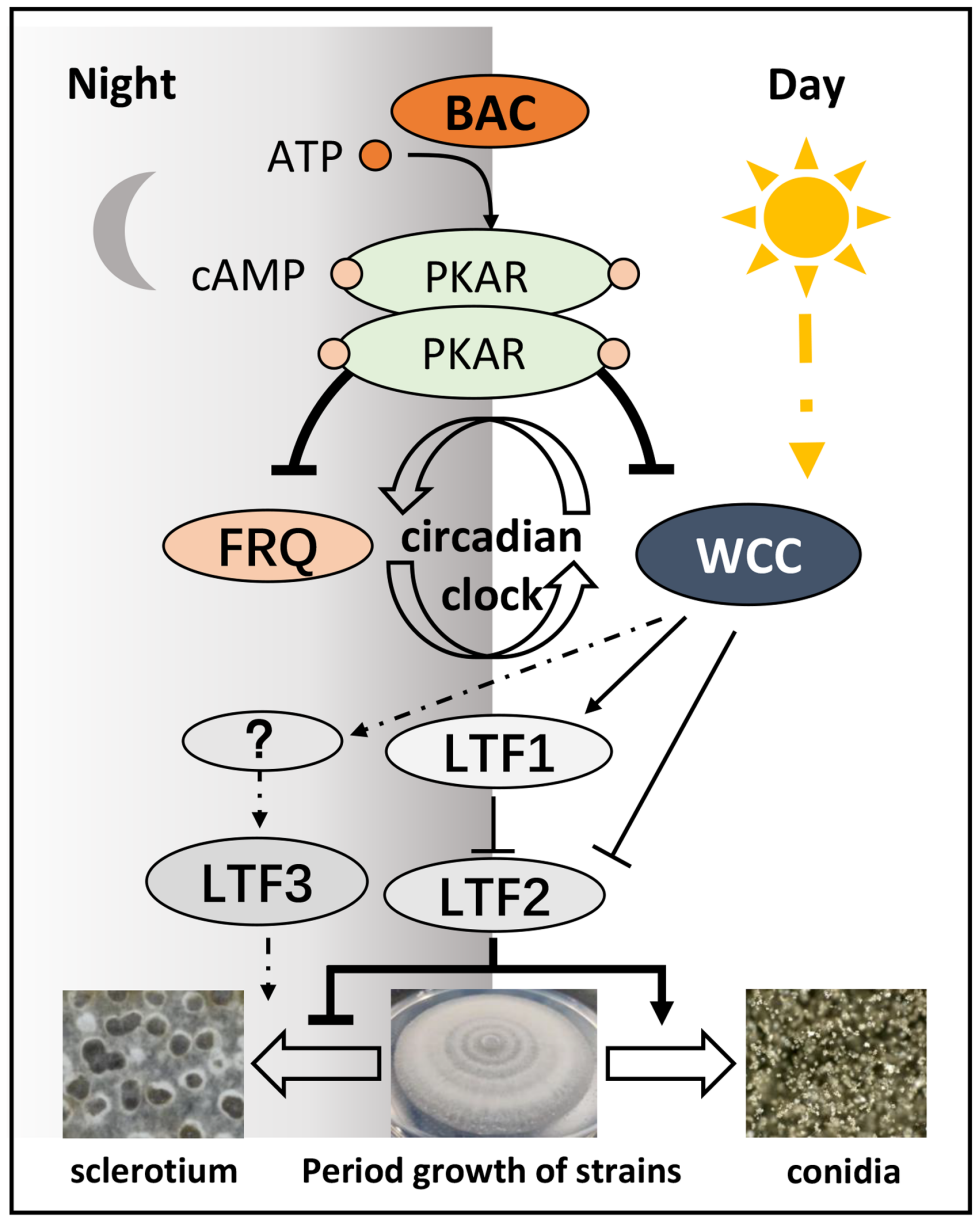
The conidiation and sclerotia formation in *B. cinerea* are regulated by the cAMP signaling pathway.

In summary, we identified BAC through the synthesis of cAMP to affect circadian clock component expression stability, and downstream genes, such as *Bcltf1*, *Bcltf2*, and *Bcltf3*, were regulated by circadian clock components to form conidia or sclerotium in response to changes in the light environment (Figure 11). This indicates that the cAMP signal, which is mediated by BAC, is very important for adjusting the survival strategy of *B. cinerea*.

## Data availability statement

The original contributions presented in the study are included in the article/Supplementary material, further inquiries can be directed to the corresponding author.

## Author contributions

YC and LX conceived the study. YC, XC, PL, WR, QZ, and YW performed the experiments. YC, YJ, PZ, HT, and LX analyzed the data and interpreted the results. YC, HT, and LX took the lead in writing the manuscript. All authors read and approved the final manuscript.

## Funding

This work was supported by the National Natural Science Foundation of China (grant nos. 31972121 and 32061133006).

## Conflict of interest

The authors declare that the research was conducted in the absence of any commercial or financial relationships that could be construed as a potential conflict of interest.

## Publisher’s note

All claims expressed in this article are solely those of the authors and do not necessarily represent those of their affiliated organizations, or those of the publisher, the editors and the reviewers. Any product that may be evaluated in this article, or claim that may be made by its manufacturer, is not guaranteed or endorsed by the publisher.

## Footnotes

^1^ http://fungi.ensembl.org/Botrytis_cinerea/Info/Index

^2^ https://www.ncbi.nlm.nih.gov/

^3^ https://www.ebi.ac.uk/interpro/

^4^ http://smart.embl-heidelberg.de/

^5^ https://services.healthtech.dtu.dk/service.php?NetPhos-3.1
