# Supplementary material for: Phosphorylation status of a conserved residue in the adenylate cyclase of Botrytis cinerea is involved in regulating photomorphogenesis, circadian rhythm, and pathogenicity
Source: Front Microbiol. 2023 Feb 15;14:1112584. doi: 10.3389/fmicb.2023.1112584 (PMC9975511; doi:10.3389/fmicb.2023.1112584)
Supplement: Supplementary file 1 [file Table_1.DOCX]

**Table S1 *Botrytis cinerea* strains and plasmid used in this study**

| **Strain** | **Gene function or Purpose** | **Origin** |
| --- | --- | --- |
| B05.10 | *B. cinerea* wild type strain | Presented by Professor Matthias Hahn of kaiserlausten University, Germany |
| *bac^S1407P^* | *B. cinerea* adenylate cyclase S1407P single amino acid mutation | Chen et al. (2020) |
| *bac^S1407A^* | *B. cinerea* adenylate cyclase S1407A single amino acid mutation | This study |
| *bac^S1407D^* | *B. cinerea* adenylate cyclase S1407D single amino acid mutation | This study |
| *bac^P1407S^* | In situ replenishment strain of *B. cinerea* adenylate cyclase S1407P single amino acid mutation | Chen et al. (2020) |
| Δ*bcwcl1* | Transcription factor/  photoreceptor BcWCL1 mutation, and BcWCL1 with its partner BcWCL2 could form the White-Collar Complex (WCC) | Zhu et al. (2018) |
| Δ*bcwcl1-com* | The compensatory strain of Δ*bcwcl1* | Zhu et al. (2018) |
