# Supplementary material for: Phosphorylation status of a conserved residue in the adenylate cyclase of Botrytis cinerea is involved in regulating photomorphogenesis, circadian rhythm, and pathogenicity
Source: Front Microbiol. 2023 Feb 15;14:1112584. doi: 10.3389/fmicb.2023.1112584 (PMC9975511; doi:10.3389/fmicb.2023.1112584)
Supplement: Supplementary file 2 [file Table_2.DOCX]

**Table S2 The List of primers used in this study**

| **Primer** | **Sequence** | **purpose** |
| --- | --- | --- |
| P1  P2 | AAGACGGTGTCGGTGGTG  GACATTGAAGGAGCATTTTTTGGGC | Primers to identify *bac* site-directed mutant |
| P3  P4 | TTCGGTCTCCGTCTGCGAATG  AAGACGGTGTCGGTGGTG | Primers to identify *bac* site-directed mutant |
| P5  P6 | GACATTGAAGGAGCATTTTTTGGGC  CAGGCAACCAGGAGTGAA | Primers to identify *bac* site-directed mutant |
| P7  P8 | AAAAGCCTGAACTCACCGC  TCGTCCATCACAGTTTGC | The identification primer of hyg |
| P9  P10 | CTGGCGGCATTATTGGT  AGACGGTGTCGGTGGTG | The identification primer of nat |
| P11  P12 | CCATGGAGGCCAGTGAATTCATGTCACTACCGTCGGTGTA  TTCATCTGCAGCTCGAGCTCTTAGTTCTGGTGAAGGGGAT | *BcpkaR* fragment, linked to vector pGAD-T7 |
| P13  P14 | TGGCCATGGAGGCCGAATTCATGAATTCGGAATCGGATCC  TATGCGGCCGCTGCAGGTCG TCAAAGCTCCACCTCATCGC | *Bcfrq1* fragment, linked to vector pGBD-T7 |
| P15  P16 | TGGCCATGGAGGCCGAATTCATGAGTAATTTCTTCAATAT  TATGCGGCCGCTGCAGGTCGTCAATTCATTCCACTCGTTT | *Bcwcl1* fragment, linked to vector pGBD-T7 |
| P17  P18 | GCAAATGGGTCGCGGATCCATGCGTACTTCAGGCTCTCTTGCAGG  AGTGGTGGTGGTGGTGGTGCTACGGTTTCGAAATCATCTT | BAC PPM and PP2C domain fragment linked to vector pet28a+ |
| P19  P20 | TGCCTCTCCCGAATTCATGAGTAATTTCTTCAAT  CGAGTCGGCCGAATTCATTCATTCCACTCGTTTT | *Bcwcl1* fragment, linked to vector pB42AD |
| P21  P22 | ATCTGTCGACCTCGAGGACTGGTGGGAGGGAGGCAC  GAGCACATGCCTCGAGGGAGCAACTTCCCAATTT | *Bcfrq1* promoter fragment, linked to vector pLaczi |
| P23  P24 | ATCTGTCGACCTCGAGCGACAGCAATCGATCGACTG  GAGCACATGCCTCGAGTGTAAGATCTGGGAATAT | *Bcltf1* promoter fragment, linked to vector pLaczi |
| P25  P26 | ATCTGTCGACCTCGAGCTTTTAGGATATCGTTTGTA  GAGCACATGCCTCGAGTACAAAGGCTTGGAGAAA | *Bcltf2* promoter fragment, linked to vector pLaczi |
| P27  P28 | ATCTGTCGACCTCGAGGAGATATACTTTTGATCAAC  GAGCACATGCCTCGAGGGTGATTGATGTGGTTGA | *Bcltf2* promoter fragment, linked to vector pLaczi |
