# Supplementary figures and images for: Phosphorylation status of a conserved residue in the adenylate cyclase of Botrytis cinerea is involved in regulating photomorphogenesis, circadian rhythm, and pathogenicity
Source: Front Microbiol. 2023 Feb 15;14:1112584. doi: 10.3389/fmicb.2023.1112584 (PMC9975511; doi:10.3389/fmicb.2023.1112584)

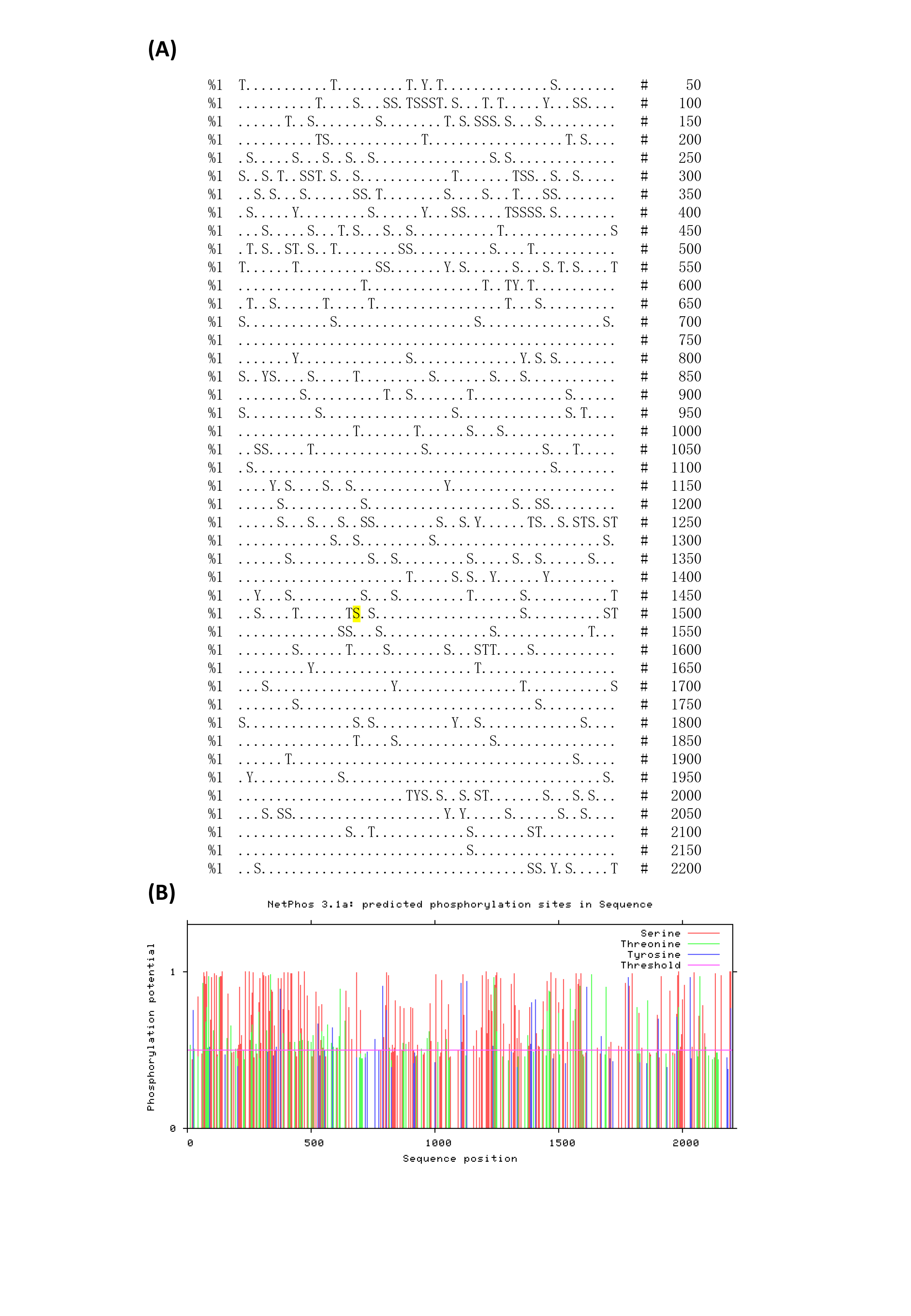

Supplement: Supplementary file 3 [file Image_1.TIF]

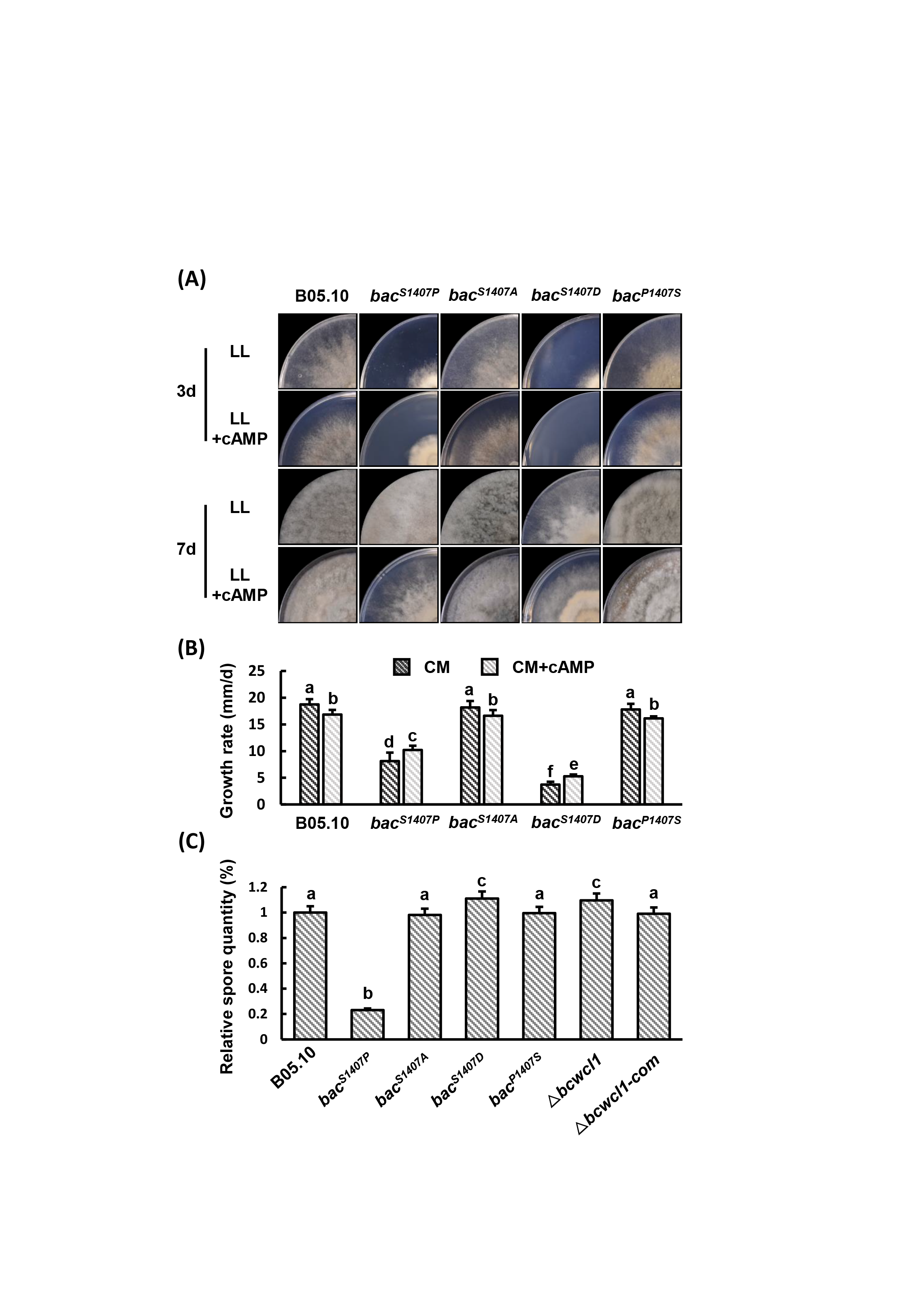

Supplement: Supplementary file 4 [file Image_2.TIF]

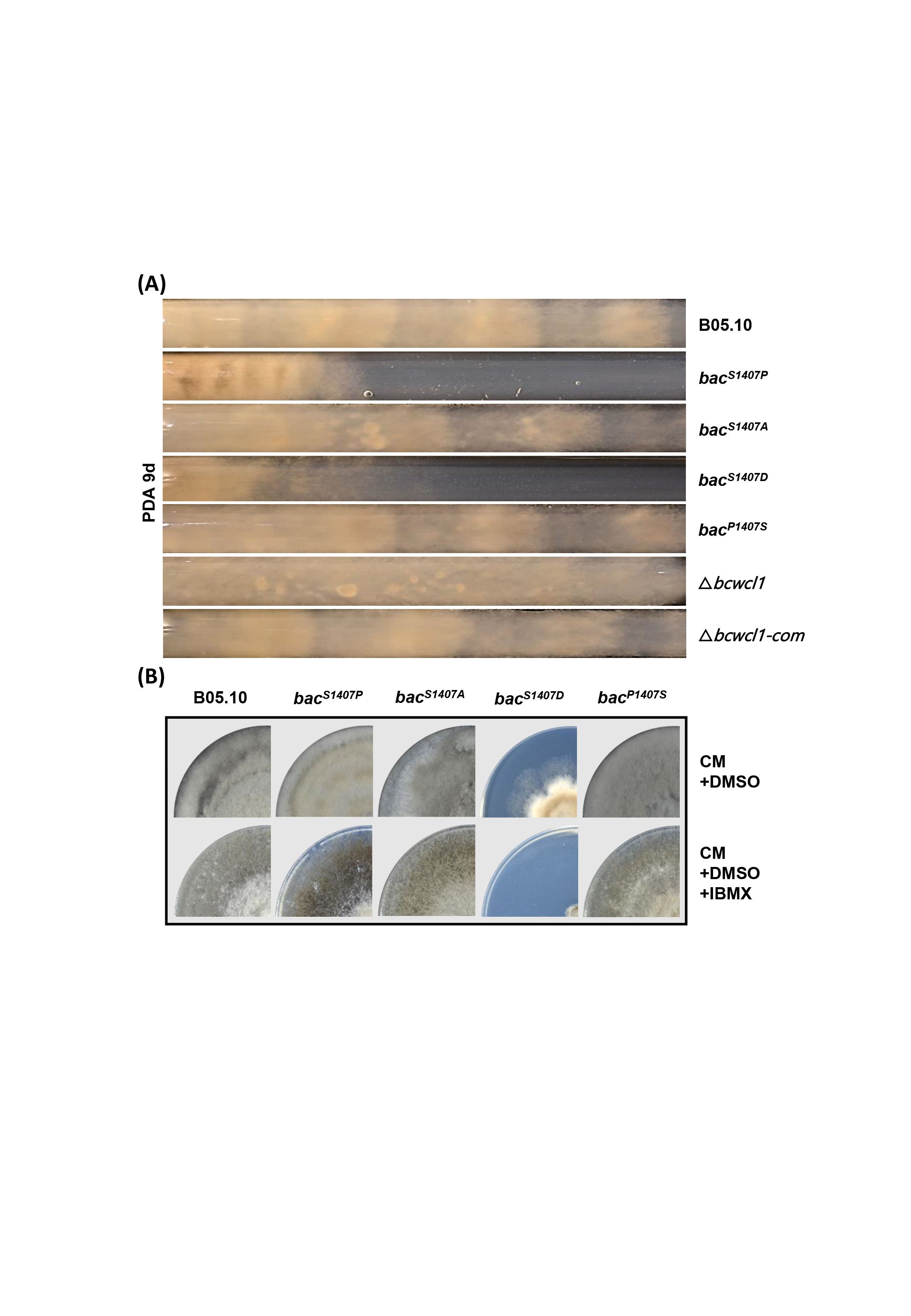

Supplement: Supplementary file 5 [file Image_3.TIF]

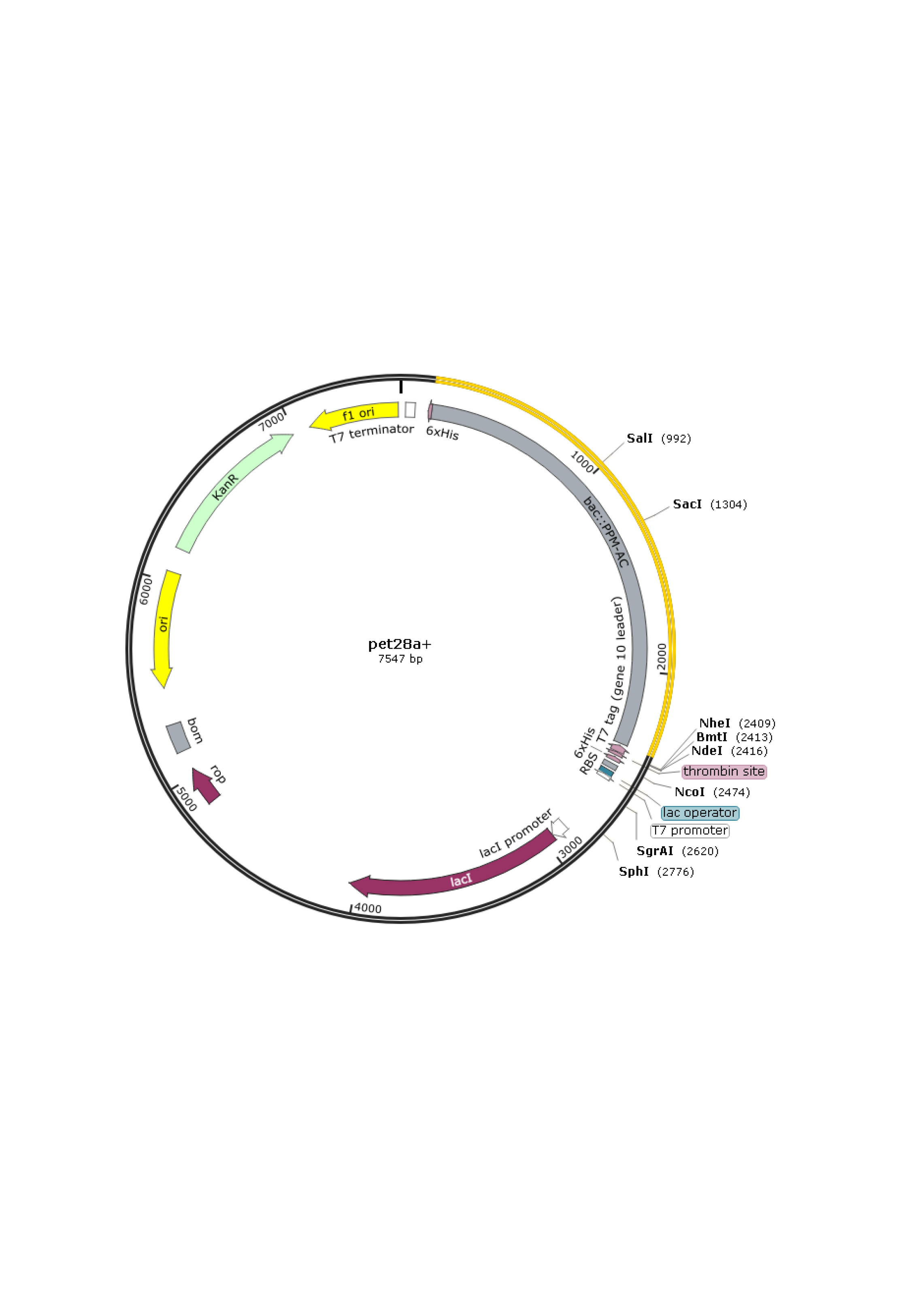

Supplement: Supplementary file 6 [file Image_4.TIF]
